# A Pelvic Pseudotumor in a Nonhemophilic Patient: An Unusual Presentation

**DOI:** 10.1155/2015/359735

**Published:** 2015-04-27

**Authors:** Mohamad Gouse, Abel Livingston, Dan Barnabas, Vinoo Mathew Cherian

**Affiliations:** Unit 1, Department of Orthopaedics, Christian Medical College, Vellore 632004, India

## Abstract

Hemophilic pseudotumor is a rare complication of hemophilia, occurring in 1 to 2 percent of individuals with severe factor VIII or factor IX deficiency. A 35-year-old male presented with a swelling in the right lower abdomen for 3 months. There was no history of trauma. Examination revealed a swelling over the right iliac fossa. Right hip showed 30° flexion deformity. Blood investigations like complete blood count, APTT, PT, bleeding and clotting time, and fibrinogen were all normal. Plain radiograph and MRI showed a lytic lesion in the right iliac wing. Excision biopsy of the swelling showed organized hematoma with a fibrous capsule suggestive of a pseudotumor. Further haematological workup like factors VIII and IX was normal. At 2 years follow-up, there was no recurrence. We report this case of pseudotumour in patient without any bleeding disorder. Such case has not been reported in literature to the best of our knowledge.

## 1. Introduction

Pseudotumors are part of a spectrum of hemophilic cysts. Hemophilic pseudotumor or hemophilic cyst is a rare complication of hemophilia, occurring in 1 to 2 percent of individuals with severe factor VIII or factor IX deficiency [1]. Pseudotumors in hemophilic patients usually involve the extremities and, less frequently, the pelvic region [2]. The pathogenesis of pseudotumors is not fully understood. These lesions are usually seen in soft tissues but occasionally occur de novo in bone or in a subperiosteal location.

We report this case of a patient without a bleeding disorder, who presented to us with an abdominal mass. The initial diagnosis was a bone tumor which later turned out to be a primary pseudotumor in a nonhemophilic patient. To the best of our knowledge, such case has not been reported in literature.

## 2. Case Report

A 35-year-old male presented with a swelling in the right lower abdomen, reportedly existing for three months. He was apparently well till three months ago, when he developed pain over the right lower abdomen, radiating down to the right lower limb. Pain increased with exertion and was partially relieved with rest and analgesics. He subsequently noticed a swelling in the right iliac region which increased in size progressively. There was no history of trauma. Examination revealed a swelling over the right iliac fossa which was smooth, immobile, and firm in consistency. He had right hip fixed flexion deformity of 30 degrees with further flexion up to 110 degrees. Routine blood investigation results were normal.

**Figure 1:**
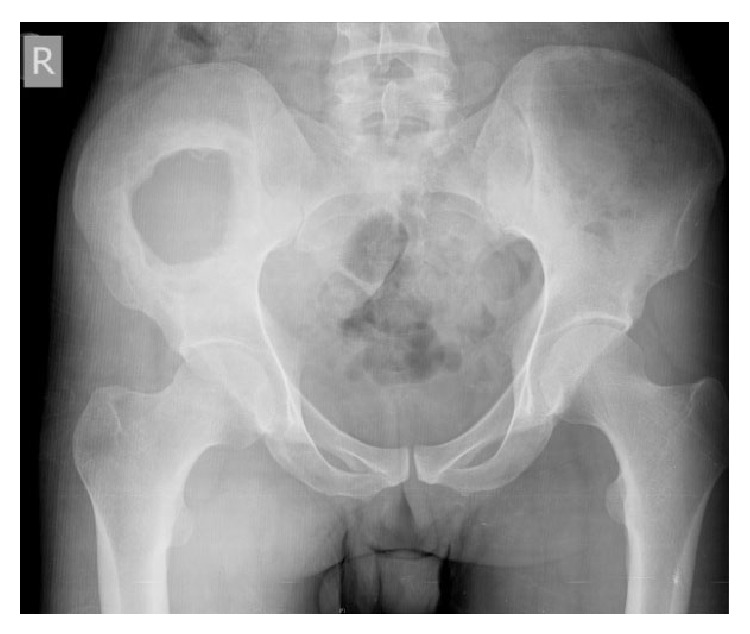
Plain radiograph showing a lytic lesion in the right iliac wing with minimal periosteal reaction.

**Figure 2:**
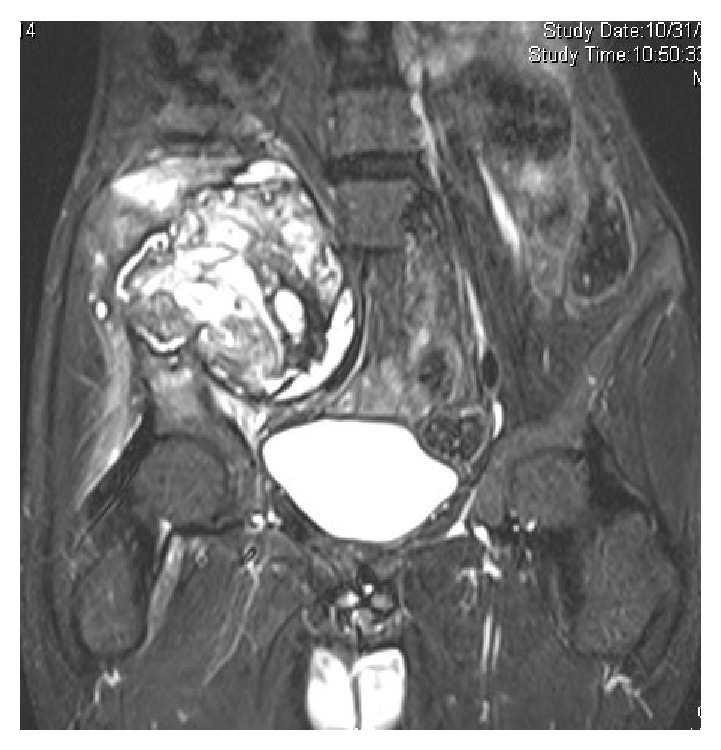
MRI T2W coronal view showing a large lytic lesion involving the right iliac bone with a large soft tissue component, with heterogeneous signal intensity with lobulated margins and hypointense capsule.

**Figure 3:**
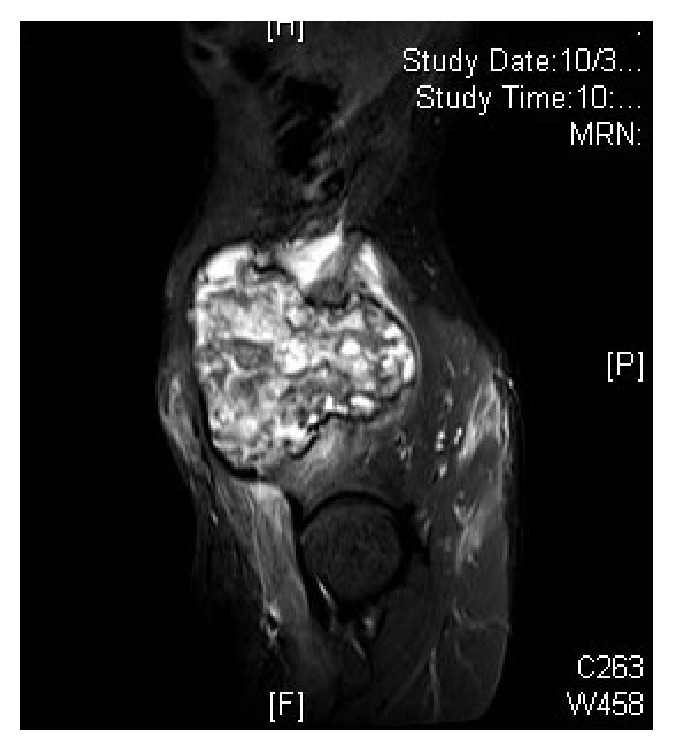
MRI T2W sagittal cuts showing dumbbell shaped mass present on either side of the right ilium.

Plain radiograph showed a lytic lesion in the right iliac wing, measuring 5 × 6 cm with minimal periosteal reaction (Figure 1). Ultrasound showed a 12.5 × 12.8 cm large solid mass in right iliac fossa with destruction of underlying iliac bone suggestive of chondrosarcoma or round-cell tumor. Bone scan was done which showed a primary lesion of the right iliac bone. MRI showed a large lytic lesion involving the right iliac bone with a large soft tissue component, with heterogeneous signal intensity with lobulated margins and hypointense capsule with no encasement of the neurovascular bundle. Involvement of the gluteal muscles and iliopsoas muscles was also noted (Figures 2 and 3). These features were more in favor of an aggressive neoplastic lesion. A trucut biopsy was done, and the sample being inadequate a repeat biopsy under ultrasound guidance became necessary. Ultrasound guided biopsy of right iliac fossa soft tissue mass lesion was done under local anesthesia which showed friable and necrotic tissue.

**Figure 4:**
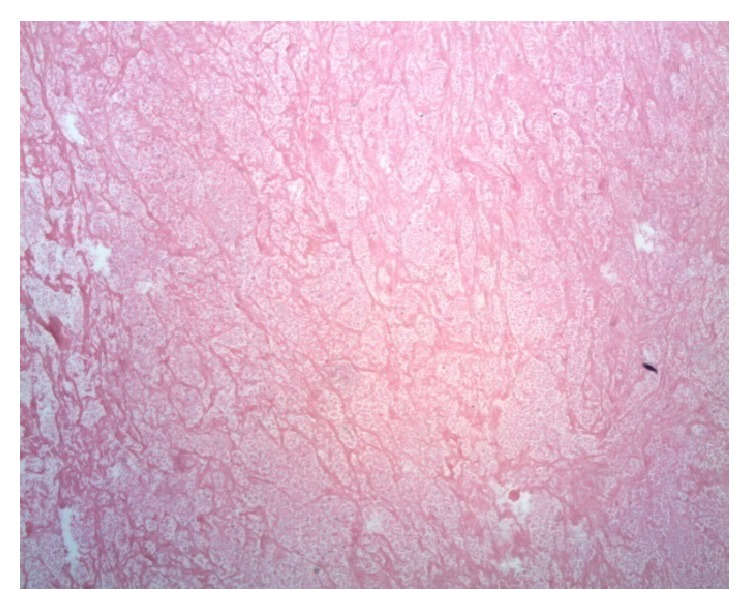
Organized hematoma with fibrin and scattered inflammatory cells, with no signs of malignant degeneration noted; the tumor consisted mainly of organized hematoma with a fibrous capsule.

Since the diagnosis was inconclusive, the patient was planned for excision biopsy of the swelling. On lateral position, the tumor was exposed. It had eroded the ilium and extended into the gluteal area in a dumbbell fashion. The well-encapsulated tumor was excised and sent for histopathological examination. Microscopically, it consisted mostly of organized hematoma, with central signs of recent haemorrhage. No signs of malignant degeneration were seen. The tumor consisted mainly of organized hematoma with a fibrous capsule (Figure 4). The pathology report confirmed diagnosis of a pseudotumor. The complete haematological workup is listed in Table 1.

Postoperatively, flexion deformity of hip improved and there was delay in wound healing due to serous collection which resolved spontaneously. At 2 years follow-up, there was no recurrence of the symptoms and the patient had returned to work.

## 3. Discussion

Hemophilic pseudotumors were first described in literature by Starker in 1918 [3]. The pathogenesis of pseudotumors is not fully understood. Proposed theories include an extension from hemarthrosis, soft tissue or subperiosteal haemorrhage, or cortical or medullary haemorrhage. Any of these mechanisms is possible when there is history of trauma [1]. However, this patient did not give any history of trauma.

The clinical presentation of the hemophilic pseudotumor will be a painless enlarging mass with episodes of acute pain due to internal bleeding [4]. The morbidity occurs in pseudotumors due to bone destruction, muscle necrosis, and local pressure effects. This patient also had similar symptoms of a slow growing mass with intermittent pain radiating to the left lower limb. Since there was no history of bleeding disorder in the past, we did not consider pseudotumor in the differential diagnosis.

On plain radiograph, the dense appearance within the haemophilic cyst is due to deposition of haemosiderin pigment. This picture is also noticed in benign, malignant, and infectious conditions. The differential diagnoses were malignant bone tumors including fibrosarcoma, plasmacytoma, malignant fibrous histiocytoma, telangiectatic osteosarcoma, metastatic disease from primary tumors in the kidney, thyroid gland, or lung and benign bone tumors such as aneurysmal bone cysts, solitary bone cysts, brown tumors, and echinococcosis [5]. The radiograph of this patient showed the osteolytic lesion with well-defined margins with no periosteal reaction, which is also the feature of a typical haemophilic pseudotumour. It is very unlikely to think of pseudotumor in a normal individual with these radiological features. Hence, there was delay in arriving at diagnosis in this patient.

In pseudotumour, ultrasonography is not helpful in detecting the bony changes but is useful in monitoring the recurrence of the tumour postoperatively [6].

MRI is the most useful modality of imaging in establishing the diagnosis of a pseudotumour [7]. But this imaging modality is nonspecific for pseudotumor if the radiologist is not aware of a bleeding disorder. In this case, there was no history suggestive of a bleeding disorder and hence malignancy was suspected.

The histopathological features of haemophilic pseudotumor consist of hematoma with dense fibrous capsule. The fibrous wall consists of collagenous connective tissue and variable amount of organized fibrous tissue [8]. The histopathology of this patient was that he also had these features but he was nonhemophilic.

All the above-mentioned clinical, radiological, and histological features were characteristic features of the typical hemophilic pseudotumor. This patient had all features of a pseudotumour, but he did not have a bleeding disorder which was confirmed with a complete haematological workup.

Stevenson and Keast had reported pseudotumor causing epistaxis in a nonhaemophilic patient with aortic valve replacement under warfarin treatment [9]. Even in hemophiliacs, the involvement of pelvis is rare [10]. To the best of our knowledge, this is the only case of a pelvic pseudotumour in a nonhaemophiliac patient. This case is presented due to its unique presentation and diagnostic dilemma.

## Key Message

The occurrence of pseudotumour in a normal patient without any bleeding disorder or trauma is extremely rare and has not been reported in the literature so far.

## Figures and Tables

**Table 1:** 

Parameters	Values
(1) Haemoglobin	8.8 g/dL
(2) Total count	13600/cumm
(3) Platelets	169000/cumm
(4) Bleeding time	2 minutes and 30 seconds
(5) Prothrombin time (PT)	11.8 secs (10.6–13.8 secs)
(6) APTT (clot based assay)	32.3 secs (28.7–39.3 secs)
(7) INR	1.11
(8) Factor VIII	52.0%
(9) Factor IX	84.9%
(10) Factor XI	66.4%
(11) Factor XIII antigen	36.1%
(12) Factor XIII activity	Normal
(13) Vwf : RCo	124%
(14) Fibrinogen	281.0 mg%
